# Misconceptions in the health technology industry that are delaying the translation of artificial intelligence technology into relevant clinical applications

**DOI:** 10.1590/0100-3984.2020.0151

**Published:** 2021

**Authors:** Fabíola Macruz

**Affiliations:** 1 Private Clinical AI Advisor, São Paulo, SP, Brazil.

**Keywords:** Artificial intelligence/supply & distribution, Industry/organization & administration, Software/trends, Delivery of health care/trends, Diagnosis, computer-assisted/trends, Inteligência artificial/provisão & distribuição, Indústrias/organização & administração, *Software*/tendências, Assistência à saúde/tendências, Diagnóstico por computador/tendências

## Abstract

There is great optimism that artificial intelligence (AI), as it disrupts the medical world, will provide considerable improvements in all areas of health care, from diagnosis to treatment. In addition, there is considerable evidence that AI algorithms have surpassed human performance in various tasks, such as analyzing medical images, as well as correlating symptoms and biomarkers with the diagnosis and prognosis of diseases. However, the mismatch between the performance of AI-based software and its clinical usefulness is still a major obstacle to its widespread acceptance and use by the medical community. In this article, three fundamental concepts observed in the health technology industry are highlighted as possible causative factors for this gap and might serve as a starting point for further evaluation of the structure of AI companies and of the status quo.

## Abstract (pt)

Há uma grande expectativa de que a inteligência artificial (IA), ao transformar a medicina, determine melhoras relevantes em todas as áreas da assistência médica, desde o diagnóstico até o tratamento. Simultaneamente, há evidências de que algoritmos baseados em IA já ultrapassaram o desempenho do ser humano em diversas atividades, como, por exemplo, na análise de imagens médicas ou na associação entre sintomas e biomarcadores com o diagnóstico e prognóstico de doenças. No entanto, a defasagem entre o potencial de desempenho das ferramentas ou aplicativos médicos que utilizam IA e sua relevância clínica prejudica bastante a utilização em larga escala desses programas de computadores. Neste artigo, três conceitos básicos da indústria de tecnologia da saúde são sugeridos como possíveis fatores causais para essa dissincronia entre desempenho e utilidade. Tal discussão pode servir como ponto de partida para uma avaliação mais profunda sobre a estrutura e *status quo* da indústria médica tecnológica atual.

## INTRODUCTION

The time may have finally come for artificial intelligence (AI), after periods of hype followed by several “AI winters” over the past decades. However, the translation of this technology into relevant and well-accepted clinical applications has been slower than expected. Specialists have pointed out a number of technical misconceptions as possible reasons for that delay: the idea that more data is all that is needed for a better model, disregarding the importance of labeling quality; the notion that an accurate model is all that is needed in order to create a useful product; and the concept that a good product is sufficient for having a clinical impact. However, little attention has been given to common structural and conceptual pitfalls in the health care industry that are also undermining the clinical relevance and quality of AI tools, as well as limiting the power of the AI revolution.

### The misunderstanding what AI technology is

The first problem is a misconception of what AI is, which leads to an underestimation of its potential. This is most probably due to the confusion between two concepts: technological innovation and digital transformation. Most of the talented, intelligent, motivated minds involved in AI-based software creation, who are daily driven by the concept of technological innovation, are completely disconnected from the concept of digital transformation. Simply put, in a technology company, technological innovation corresponds mostly to new processes or tools created with the purpose of simplifying, enhancing or replacing pre-existing approaches. Less commonly, technological innovation produces new processes or tools for new demands. Whether such innovations update an old way of doing things or create a new way, their impact, in terms of augmenting the efficiency or accuracy of a task, is limited to their direct users. In contrast, digital transformation is a much broader concept: it is the process of changing an organization to allow the integration of digital technology into all areas of a business, fundamentally changing how the organization operates and delivers value to its customers. It is also a cultural change that requires organizations to continually challenge the status quo, experiment, and get comfortable with failure.

The magnitude of the requirements for AI development, which includes a huge dataset, as well as considerable computer power and collaboration efforts, and the magnitude of the goals of AI, which include substantial robustness, expressive generalizability, and a broad consumer base, put AI in a very different category than previous innovative technologies and bring AI closer to digital transformation. In fact, over the past years it has become practically impossible to talk about AI without mentioning digital transformation; at the same time that AI is one of the central enablers of the digital transformation journey, it is greatly dependent on the latter.

However, because digital transformation depends on a much higher level of investment and commitment than does technological innovation, most companies prefer to see AI as the latter and not review concepts such as leadership and culture or the long-standing business processes that they were built upon. After all, it is easier to invest in a single product than in a whole company. The main problem with that strategy is that there are no shortcuts. In the health technology industry, digital transformation not only allows improvement of the customer experience, friction reduction, increased productivity, and greater profitability but is also imperative for innovation and for the translation of AI technology into truly disruptive clinical applications.

### The acceptance of superficial internal process optimization instead of profound internal cultural transformation

When the importance of digital transformation is appreciated, another misconception might pose a challenge for relevant clinical innovation: the confusion between internal cultural changes and internal process optimizations.

A cultural transformation requires taking an introspective look at the company and making changes to shape policies, commitments, processes, and behaviors so they reflect the values and beliefs of the employees. Among the keys to a successful cultural transformation are full engagement from all levels of leadership throughout the organization and a good relationship between leadership and the workforce. In regard to the first, leaders play a vital role in modeling and coaching the desired behaviors that will permeate the company. Without a commitment from leaders to transform the culture, employees are not likely to make lasting behavioral changes on their own. In regard to the second, a good relationship between leadership and the workforce is not as trivial as it may sound. It is not unusual for there to be a disconnect between leadership and workers, whether in terms of communication, expectations, agendas, or strategic vision, especially in traditional companies that create innovation hubs at their main headquarters. In addition, smaller companies can easily become a threat to larger, high-profile brands, and the latter can view cultural transformation as too risky from a reputational point of view, which suddenly turns the corporate board into a significant barrier to progressive business. This barrier is overcome only by hiring new, creative, intrinsically motivated staff and giving them the freedom to do what they do best.

On the leadership side, it takes boldness to invest in the process of cultural transformation, especially when research suggests that only 12% of companies that undertake cultural transformation achieve what they set out to accomplish^(1)^. It also takes a proactive approach and resilience to propose a transparent democratic environment that stimulates inclusion and diversity, as well as intellectual and communicative freedom. On the workforce side, it takes a significant amount of effort to improve collaboration across functional and departmental lines, to decrease bureaucracy, and to come up with solutions to make workflows more efficient.

By not reviewing concepts and work practices, challenging procedures and methodologies, addressing inequality, and favoring inclusion, corporations are doing more than just holding back cultural changes; they are actually reducing the generalizability of AI medical software, which can ultimately expose the user population to avoidable risks.

Because AI tools replicate and potentiate biases existing in our society and in the corporative environment, the need for an unbiased setting becomes more relevant in the era of AI. A good example of this is the fact that gender-biased companies are less likely to acknowledge gender inequality in datasets, differences in the prevalence and prognosis of the disease in question, and potential gender-related variabilities in the performance of the AI-based software. What would lead to a final product with less acceptance by female consumers in the standard technology marketplace now leads to a gender-biased neural network with poorer performance in women. This alone should encourage behavioral changes, including the acknowledgment of minorities previously ignored.

In the age of AI, nurturing a democratic, collaborative, inclusive environment that will lead to a generalizable clinical tool should not be a choice; it should be legal responsibility. Unfortunately, we know that this is far from reality; most companies are still highly biased regarding gender, age, race, nationality, ethnicity, religious background, and sexual orientation and not very enthusiastic about investing in cultural transformation.

### The importance of having a medical specialist leading the development team

Most health technology organizations are a battlefield between three groups of people: the business-oriented people, the life-science people, and the exact-science people. It is still a challenge to create a cross-functional team, in which differently skilled groups approach the AI challenges as equal partners.

Creating the type of organization in which all groups genuinely lead together and deal with the mismatch between what they want the technology to accomplish and what it can in fact accomplish, might require hiring a new set of “bilingual” open-minded specialists; that is, specialists who are willing to form a collaborative team and to entertain perspectives offered by specialists from other fields, even if those perspectives are very different from their own. Workers who can bridge the gaps between differently skilled people and speak intelligently on all sides are rare. Therefore, in most AI companies, there is a significant skew toward one group that establishes the strategic perspective and dictates the final agenda. Unfortunately, that agenda is rarely focused on the clinical purpose and on the use case.

It is not unfair to state that, in a health technology organization, the clinical team is usually seen as a complement and has little involvement in the AI software development lifecycle. The vast majority of clinicians are limited to selecting cohorts, labeling imaging data, or doing project management. This makes us wonder who is actually identifying the clinical challenges, finding alternatives to address them, providing the technical medical insights, and ensuring the quality of the AI-based software.

Together with companies, specialists are partially to blame: they should be less reluctant to engage with the new AI health technology world. The ultimate goal of health care AI should be to humanize the field of medicine and make it more comprehensive. That will require human activism and greater engagement on the part of the medical community in standing up for the best interest of patients. To date, the opposite has been observed: passivity on the part of clinicians, similar to that seen during the creation of other major innovations in the health care field, such as the electronic medical records. This lack of proactivity and engagement by clinical personnel in relation to AI technology may lead to an outcome similar to that observed back then; that is, the creation of a tool that favors time savings and financial efficiency of the workflow over clinical competence and coherence, as well as over emotional engagement between doctors and patients.

The failure to recognize the value of medical specialists by the organizations and the silence from the medical community have several consequences. The immediate consequence is the underuse of the clinical knowledge, which results in the creation of tools that are less clinically relevant and that integrate less organically into the medical routine. Among the long-term consequences are the underutilization of the capacity of AI to humanize health care and the potential harm to the patients that count on the expertise of a specialist to receive a proper diagnosis and the appropriate treatment. As stated by Francis W. Peabody in his article^(2)^ and quoted by Eric Topol in his latest book^(3)^, “…the secret of the care of the patient is in caring for the patient”, and few people will ever care more about patients than do physicians.

The recognition of the points made here is the first step on a long road. Only through the pursuit of digital transformation, the review of corporate cultures, and the deep engagement of medical specialists in the developmental process of AI-based tools will the translation of AI technology into relevant clinical applications reach its full potential. Thus, the AI revolution will be able to put the “care” back into health care.
